# When Stiffness Matters: Mechanosensing in Heart Development and Disease

**DOI:** 10.3389/fcell.2020.00334

**Published:** 2020-05-25

**Authors:** Roberto Gaetani, Eric Adriano Zizzi, Marco Agostino Deriu, Umberto Morbiducci, Maurizio Pesce, Elisa Messina

**Affiliations:** ^1^Department of Molecular Medicine, Faculty of Pharmacy and Medicine, Sapienza University of Rome, Rome, Italy; ^2^Department of Bioengineering, Sanford Consortium for Regenerative Medicine, University of California, San Diego, San Diego, CA, United States; ^3^PolitoBIOMed Lab, Department of Mechanical and Aerospace Engineering, Politecnico di Torino, Turin, Italy; ^4^Tissue Engineering Research Unit, “Centro Cardiologico Monzino,” IRCCS, Milan, Italy; ^5^Department of Maternal, Infantile, and Urological Sciences, “Umberto I” Hospital, Sapienza University of Rome, Rome, Italy

**Keywords:** cardiac regeneration, mechanosensing and regulation, cardiac tissue engineering, tissue modeling, stiffness

## Abstract

During embryonic morphogenesis, the heart undergoes a complex series of cellular phenotypic maturations (e.g., transition of myocytes from proliferative to quiescent or maturation of the contractile apparatus), and this involves stiffening of the extracellular matrix (ECM) acting in concert with morphogenetic signals. The maladaptive remodeling of the myocardium, one of the processes involved in determination of heart failure, also involves mechanical cues, with a progressive stiffening of the tissue that produces cellular mechanical damage, inflammation, and ultimately myocardial fibrosis. The assessment of the biomechanical dependence of the molecular machinery (in myocardial and non-myocardial cells) is therefore essential to contextualize the maturation of the cardiac tissue at early stages and understand its pathologic evolution in aging. Because systems to perform multiscale modeling of cellular and tissue mechanics have been developed, it appears particularly novel to design integrated mechano-molecular models of heart development and disease to be tested in *ex vivo* reconstituted cells/tissue-mimicking conditions. In the present contribution, we will discuss the latest implication of mechanosensing in heart development and pathology, describe the most recent models of cell/tissue mechanics, and delineate novel strategies to target the consequences of heart failure with personalized approaches based on tissue engineering and induced pluripotent stem cell (iPSC) technologies.

## Introduction

In recent years, the assessment of biomechanical-dependent molecular machinery has become a new insightful approach to decipher cellular dynamics inside tissues, with implications in morphogenesis, tissue renewal, and pathology progression. For example, mechanical-dependent coordination of tissue growth is one of the major determinants in either preimplantation or postimplantation embryonic patterning (Bredov and Volodyaev, 2018), as well as in pathologic evolution such as cancer (Papalazarou et al., 2018). Moreover, progenitor cells differentiation has been directly linked to compliance of the extracellular matrix (ECM) (Engler et al., 2006) or to the topography and characteristics of the substrate in which they are cultured. Similarly, when cells are confined onto geometrically defined adhesions surfaces (McBeath et al., 2004; Nelson et al., 2005), aligned in specific patterns onto microgrooved structures (Downing et al., 2013), or exposed to cyclic strain (Ugolini et al., 2016), mechanical stress sensed by the cytoskeleton results in a broad variety of cellular responses such as differentiation, proliferation, or maturation. However, what links mechanical stimuli to cell biology and how mechanical pathways are transduced inside the cell to activate proliferation and/or alternative differentiation pathways are still not fully understood. A greater discernment of these mechanisms is of fundamental importance in order to better understand the development and progression of various cardiac diseases in which a dysregulation of the mechanosensing-mediated pathways are involved. Similarly, it is now evident that the same molecular mechanisms are also involved in the maturation of a functional tissue, both *in vitro* and *in vivo.* They should be further considered when trying to engineer and develop *in vitro* new cardiac tissue, for both regenerative medicine and research applications. In this review, we will address the latest implication of mechanosensing in heart development and pathology, describe the most recent models of cell/tissue mechanics, and delineate novel strategies to target the consequences of heart failure with personalized approaches based on tissue engineering and induced pluripotent stem cell (iPSC) technologies.

## Cell Mechanics and Heart Development/Disease

### From Cytoskeleton Tensioning to Mechanical Sensing and Gene Expression Programming

Mechanical cues are converted into biochemical stimuli by connections established between the cytoskeleton [a network of protein filaments composed of microtubules (MTs), microfilaments (MFs), and intermediate filaments (IFs) involved in many cellular functions because of their role in, e.g., cell shaping, migration, and intracellular architecture conditioning] and the external ECM through focal adhesion contacts and multi-protein structures connecting the actin bundles to the ECM. Among all the proteins present in focal adhesions, integrins are transmembrane heterodimeric receptors, composed of α and β subunits, which bind to the ECM and activate different signal transduction within the cells. Similar to receptor–ligand interactions, mechanical signaling originates from receptor-mediated nucleation of adhesions structures, and further organization of intracellular cytoskeleton with consequent force generation (from intracellular or external stimuli) and intracellular mechanical transduction (Burridge and Wittchen, 2013; Brouhard and Rice, 2018). Downstream of mechanical activation (e.g., increasing force generation by cells attached to substrates with increasing stiffness) is the activation of gene transcription signaling that may have consequences for alternative differentiation of cells with progenitor characteristics. Typical is the example of mesenchymal cells that were shown to differentiate into adipose or osteogenic cells depending on the compliance of the ECM (Engler et al., 2006). This differentiation process occurs by engaging signaling cascades, for example, the so-called Hippo pathway (Piccolo et al., 2014), whose activity is directly dependent on the tension of the cytoskeleton, determining the reversible nuclear translocation of the Yes-associated protein and transcriptional activator with PDZ-binding motif (YAP/TAZ) transcription complex (Dobrokhotov et al., 2018; Dasgupta and McCollum, 2019; Pocaterra et al., 2020).

Mechanical signaling may also determine longer time effects and permanent programming of cell phenotype through specific setting of the epigenome. For example, experiments performed with hydrogels with defined compliance showed that exposure to a stiff three-dimensional (3D) environment modifies permanently gene expression with permanently established “mechanical memory” effects (Yang et al., 2014) that cannot be reversed even by shifting cells into an environment with higher compliance. In line with these evidences, it was observed that forcing cells to acquire specific geometries determines permanent changes in epigenetic marks that likely translate in long-term gene activation/repression at the chromatin level (Downing et al., 2013).

The link between intracellular mechanical signaling and permanent epigenetic changes may depend on the direct effects that generation of intracellular forces has on the opening and closing of the nuclear pores and the accessibility of the chromatin to transcriptional machineries due to long-range interactions with proteins of the nuclear envelope. For example, it was recently observed that connection of the contractile cytoskeleton to the nuclear lamina is important to promote nuclear straining and translocation of the YAP/TAZ complex by physical opening of the nuclear pores independently of the Hippo signaling (Elosegui-Artola et al., 2017; Lomakin et al., 2017). The structure of the chromatin may itself result from generation of intracellular forces due to peculiar conformation of the surrounding environment. For example, confinement of cells into specific geometric patterns resulted in topological chromatin rearrangement due to force-dependent distribution of epigenetically active enzymes [e.g., histone deacetylases (HDACs)], and this may cause permanent opening/compaction of the chromatin in specific gene loci (Jain et al., 2013). This intriguing possibility seems to be linked to the association existing between chromatin and components of the nuclear envelope (e.g., lamins), which, other than a structural function in the nucleus, may also have mechanical transduction and topological insulation functions (Stephens et al., 2018). Recently, Cho et al. (2019) demonstrated that nuclear lamina regulates and reduces the nuclear rupture and leak of DNA repair factors, suggesting a role of the nuclear lamina as a “cushion” potentially protecting from cytoskeleton-dependent nuclear mechanical stress. Together, these evidences support an integration of mechanical forces into the wider control mechanism of cell identity and function and establish spatial and mechanical criteria for correct tissue development and homeostasis.

### Mechanosensitive Control of Heart Development and Growth

Biophysical regulation of chromatin organization appears to be important also in lineage specification and pathway activation from the earliest stages of embryonic development. For example, it has been hypothesized that nuclear translocation of the YAP/TAZ transcriptional complex in response to straining of the cells outside the forming blastocysts may establish the first separation between the embryonic and extraembryonic lineages in preimplantation mammalian embryos (Nishioka et al., 2009; Biggins et al., 2015). On the other hand, cell-to-cell contacts in the inner cells activate the Hippo pathway resulting in the phosphorylation and inactivation of the YAP/TAZ, thus preventing the translocation into the nuclei and allowing pluripotency markers to be expressed (Nishioka et al., 2009; Biggins et al., 2015). This straining effect is likely related to the establishment of the apical domain of the presumptive trophectodermal cells, resulting in molecular determination of the first extraembryonic lineage by cell positioning outside the forming blastocyst and induction of pluripotency in its interior domain (Korotkevich et al., 2017).

Development of the heart is one of the first morphogenetic events occurring in the developing embryos with a complex series of cell migrations and phenotypic transformations also involving mechanical cues. Although we redirect to more specific reports describing the various lineages contributing to the formation of the heart (Meilhac and Buckingham, 2018), it is important to highlight that the origin of the coordinated heartbeat arising in the cardiac tube before its primary looping may have a pure mechanical basis rather than an electromechanical basis. This hypothesis emerges from studies showing that perturbations of mechanosensitive Ca^2+^ channels expressed by cardiac progenitors (Tyser et al., 2016) are determined by a progressive increase in the matrix stiffness leading to a coordinated beating of the newly differentiated myocardial cells (Majkut et al., 2013, Majkut et al., 2014; Chiou et al., 2016) before the onset of electromechanical coupling.

Mechanical cues seem to be profoundly involved in controlling myocyte proliferation and hypertrophy after birth. At this stage, a dramatic increase in the pumping efficiency of the heart is necessary to compensate cessation of the embryonic shunts redirecting circulation of the oxygenated blood from the placenta to the lungs, and the transition from a quasi-zero to a normal gravity level (Kennedy-Lydon and Rosenthal, 2017). The increase in mechanical performance requires a structural and functional maturation of the myocytes, with the conversion from the fetal to the adult structure of the sarcomeres (Ehler, 2016), a change in shape from polygonal to rod-like, and a sudden reduction of their proliferation with an increase in their volume. In parallel, sequestering of YAP by the dystrophin/dystroglycan complex (DGC) due to Hippo pathway-dependent phosphorylation acts as a negative feedback loop preventing further myocyte proliferation (Morikawa et al., 2017). Mechanical maturation of the heart, finally, does not only involve maturation of the contractile cell mass. In fact, the cardiac matrix undergoes a significant stiffening from a value <0.2 kPa (Young modulus) of the cardiac tube, to a value >20 kPa at birth (Majkut et al., 2013, Majkut et al., 2014; Chiou et al., 2016), when it reaches its physiological stiffness. Interestingly, a coordinated control of cardiac myosin expression in myocytes and collagen I deposition by cardiac fibroblasts (CFs) orchestrates maturation of the tissue, preparing it for the increase in mechanical load occurring at birth (Majkut et al., 2014). The connection of myocytes to the surrounding matrix through costameres establish a very sensitive feedback mechanism controlling mitotic block other than cellular mechanical integrity (Sessions and Engler, 2016), and perturbation of this system could be amenable to induce cardiac regeneration. In line with this hypothesis, it was observed that administration of Agrin, a specific component, in the neonatal cardiac matrix promotes dissociation of YAP from the DGC complex and myocyte cell cycle re-entry (Bassat et al., 2017). Because administration of drugs with a matrix softening activity prolongs the cardiac regeneration period in neonatal mice (Notari et al., 2018), it is tempting to speculate that modulating mechanical properties of the myocardial matrix could be a viable strategy to promote heart regeneration in adulthood.

## Cell and Tissue Level Mechanical Modeling: Molecular and Subcellular Level Modeling

**FIGURE 1:**
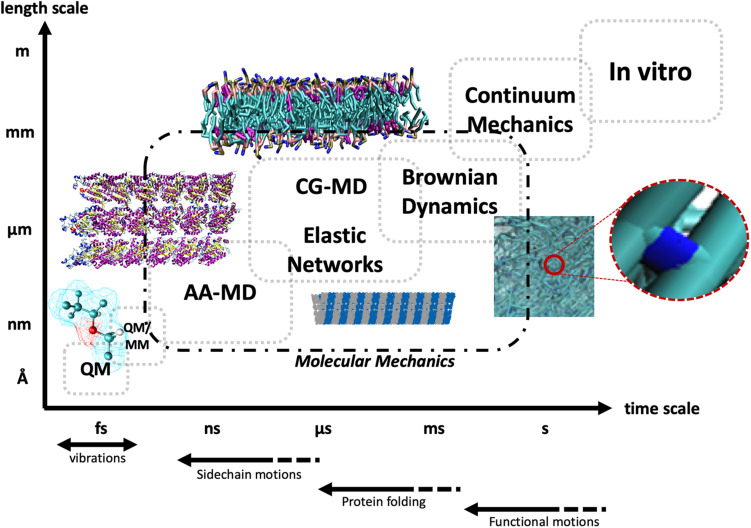
Schematic representation of the different timescales and length scales typically accessible to different computational and experimental techniques, starting from quantum mechanics (QM) and hybrid quantum mechanics/molecular mechanics (QM/MM), and progressively moving up the scales to all-atom molecular dynamics (AA-MD), coarse-grained MD (CG-MD), and elastic networks, up to Brownian dynamics and continuum mechanics, and at the top right *in vitro* approaches. The dashed line encompasses techniques deploying the principles of molecular mechanics. The bottom arrows highlight the typical timescales of different types of biological motions.

The pathways of mechanotransduction, that is, the cascade of events originating from mechanical cues eventually leading to biological responses, are known to differ considerably both between different cellular phenotypes and between distinct mechanical stimuli (Omens, 1998; Humphrey, 2001). Historically, the molecular players of mechanotransduction were categorized into the following groups (Ingber, 1997): (1) mechanical-signal-responding ion channels, (2) integrin receptors, (3) protein complexes linking the integrin receptors to the cytoskeleton, and (4) cytoskeleton components, that is, MTs, IFs, and actin MFs. Each one of these players, synergistically and sequentially involved in transmitting mechanical signals from the ECM to the inside of the cell, has benefited from new insights provided by computational theories at various length scales and timescales. Among these theories, computational molecular modeling has gained great momentum in the past 20 years, as a theoretical set of methodologies able to predict conformational and thermodynamic properties of biological building blocks and macromolecular super-assemblies by means of models characterized by atomic or quasi-atomic resolution. In this context, molecular dynamics (MD) simulations have been widely used in the last decades to obtain new insights into biological functions of proteins by studying such molecular systems at the atomistic level (Soncini et al., 2007; Lepre et al., 2017; Grasso et al., 2018, 2019a,b). After McCammon et al. (1977) investigated the dynamic behavior of small proteins on the basis of MD simulations, the method was widely employed for helping experimental design and data rationalization, with the drawback of an accessible timescale in the order of several hundreds of nanoseconds up to a microsecond (Abraham et al., 2015; Hollingsworth and Dror, 2018), in contrast with the generally much larger timescale encompassing protein function (nanoseconds up to seconds). As a matter of fact, the choice of the proper computational simulation approach is usually related to the timescale to be investigated, the size of the molecular system, and also the properties under investigation. The mechanics of large protein structures is still computationally inaccessible by all-atom MD (AAMD), which, as the name suggests, is based on the explicit modeling of all atoms of the simulated system and all the bonded and non-bonded interactions thereof. Thus, researchers working in the field tried to develop simulation approaches requiring less computational power (Chu and Voth, 2005, 2006, 2007; Marrink et al., 2007; Monticelli et al., 2008; Pfaendtner et al., 2010; Deriu et al., 2012; Bidone et al., 2015). Some of these methods are based on simplified models for describing the protein structure, such as normal mode analysis (NMA) of elastic network models (ENMs) used as a coarse-grained (CG) approach for protein dynamics (Tama et al., 2000; Tama and Sanejouand, 2001; Li and Cui, 2002; Eom et al., 2007; Tatke et al., 2008; Yang and Chng, 2008; Deriu et al., 2010; Cifra et al., 2015). These techniques represent a valid computational alternative to atomistic simulations for understanding the mechanics of large proteins. All these approaches, both atomistic and non-atomistic (Figure 1) will be discussed for each of the aforementioned biological players of mechanotransduction, starting from the cell–ECM contact and conceptually moving downstream the mechanotransduction pathway toward the cytoskeleton.

### Mechanosensitive Ion Channels

From a cellular perspective, effective translation of mechanical cues from the environment into appropriate biological responses requires a competent transduction pathway connecting the ECM to subcellular structures. For this reason, contacts between the cell and the ECM are being increasingly investigated both *in vitro* and through the application of the tools provided by computational molecular modeling, given the intimate link between mechanotransduction and protein conformational changes (Hoffman et al., 2011). Mechanosensitive ion channels (MSCs), sometimes referred to as stretch-activated channels (SACs), being located at the interface between the cell membrane and the surrounding environment, are a first candidate for the transduction of mechanical stimuli, with functional implications on a variety of biological processes including, for example, the mechano-electric feedback loop in cardiovascular physiology, where force is known to significantly alter ion conductance (Teng et al., 2014). SAC opening and closing dynamics in response to mechanical stress have been investigated both *in vitro* and *in silico* (Kizer et al., 1997; Sachs, 2010) for a variety of cellular phenotypes, and in this context, computational molecular modeling is providing valuable insights into the molecular mechanisms behind the response to mechanical stimuli. Examples of this include computational studies of the bacterial mechanosensitive channels, which are commonly categorized into mechanosensitive channels of small conductance (MscS) and mechanosensitive channels of large conductance (MscL). Sotomayor and Schulten (2004) investigated the dynamics of MscS through MD simulations both restrained to its crystallographic state and unrestrained: in the former case, the open conformation was preserved, and intermittent permeation of water was observed, whereas in the latter, unrestrained case, the presence of local surface tension was shown to mechanically induce widening of the pore. Elmore and Dougherty (2003) computationally investigated the effect of lipid membrane composition on MscL dynamics, highlighting a conformational change in the channel’s C-terminal region depending on membrane lipid composition and structural rearrangements following the thinning of the bilayer in response to mechanical tension. The gating mechanism of MscL has been further evaluated computationally by Gullingsrud et al. (2001) through the use of steered MD (SMD), where the application of lateral tension mimicking the bilayer environment subjected to mechanical stress allowed for the characterization of the dynamics of the single molecular events leading to the transition from the closed state to the first open state, in good agreement with experimental data (Sukharev et al., 1999; Kloda and Martinac, 2001). More recently, Sawada et al. (2012) identified a single residue as the main responsible for transmitting the force from the membrane bilayer to the rest of the channel, thereby characterizing the chain of events eventually leading to the first stage of channel opening, with the calculated energy difference between the closed and first conductance states in agreement with experimental data. Overall, the last two decades saw the increasing application of computational modeling techniques to MSCs, effectively shedding light on those molecular events that from the application of mechanical stimuli lead to structural rearrangements of the channels and eventually to the conformational switch to the open state. Given the crucial role of ion-channel mechanics and dynamics in the regulation of cardiovascular function, this approach is promising and expected to provide insights into the causative relationships between the cell’s mechanical environment and functional consequences in healthy and pathological myocardial conditions.

### Integrin Receptors

Integrins are cell adhesion heterodimers composed of two glycoprotein subunits named α and β, each consisting of an extracellular portion terminating with a globular shaped head, a multidomain “leg,” a transmembrane helix, and a cytoplasmic tail region. A total of nine types of β subunits and 24 types of α subunits exist, combining to form functionally distinct heterodimers (Moreno-Layseca et al., 2019). They constitute a well-known contact point between the cell and both the ECM and adjacent cells, and they have been extensively studied in terms of their mechanical properties in the context of transmitting mechanical signals downstream of focal adhesions, which are supramolecular contact points between the cell and ECM enabled by the clustering of adhesion-mediating proteins. Integrins generally bind to specific components of the ECM, which bear a distinct integrin-binding domain consisting in an Arg-Gly-Asp (RGD) triplet. The most common substrates for human integrins are laminins (α3β1 α6β1 α7β1, α6β4, α1β1, and α2β1), collagens (α1β1, α2β1, α10β1, α11β1, αvβ8, and α9β1), and fibronectin (α5β1 α8β1 αvβ1, αvβ3, αIIbβ3, αvβ6, αvβ8, α4β1, and α4β7) (Moreno-Layseca et al., 2019). The location and accessibility of RGD domains thus play a pivotal role in integrin binding, and the exposure of buried RGD triplets as a result of mechanical stress arguably constitutes a first path for mechanotransduction. Different models of the integrin activation process have been proposed. A first model postulates that inactive integrins are in a bent state, with the heads facing the cell membrane. Events such as talin binding in the intracellular region are then thought to lead to a switchblade-like motion, in which the head swings outward toward the extracellular space, thus activating the integrin (Takagi et al., 2002). A second model explained integrin activation as a consequence of the vertical sliding of the stalks in response to the same intracellular binding events (Xiong et al., 2003). Perhaps most importantly, both models explained integrin activation as a function of conformational changes enhancing the affinity of the globular heads for their extracellular ligands. Molecular modeling methods have been deployed to investigate the dynamics of integrin conformational change and activation, for example, the swing-out motion of the hybrid domain in β3 integrins. Indeed, MD and SMD simulations allowed to describe the allosteric pathway leading to hinge-angle opening in α5β3 integrins at the atomistic scale (Puklin-Faucher et al., 2006), also highlighting how RGD-sequence-containing ligand binding and ligand-dependent force application accelerate the conformational alterations (Puklin-Faucher and Vogel, 2009). The conformational dynamics of the β3 integrin headpiece hybrid domains have also been investigated by Gaillard et al. (2009) through MD and NMA, (1) underlining the low-frequency nature of the conformational transition involving the I-like and hybrid domains, (2) detailing the role of the α1 and α7 helices in the conformational transition, and (3) linking local conformational alterations of the identified regions to wider-scale structural rearrangements, allowing for integrin activation. Further studies hinted, again using targeted MD and ENMs, at common structural changes of the I-like domain essential for β3 integrin activation (Matsumoto et al., 2008; Provasi et al., 2009) and detailed the nature of the transition from inactive to active state (Chen et al., 2011). In summary, given the central role of integrins in mediating cell–cell and cell–ECM contacts and their potential as pharmacological targets (Paulhe et al., 2005), and because physiological cardiovascular function is intrinsically liked to correct mechanical coupling between its cellular and extracellular components, computational molecular modeling applied with specific methodological endpoints to the characterization of integrin-receptor mechanics and dynamics shall prove a valuable approach for the investigation of mechanotransduction, also in the context of cardiovascular function.

### The Integrin–Cytoskeleton Coupling

**FIGURE 2:**
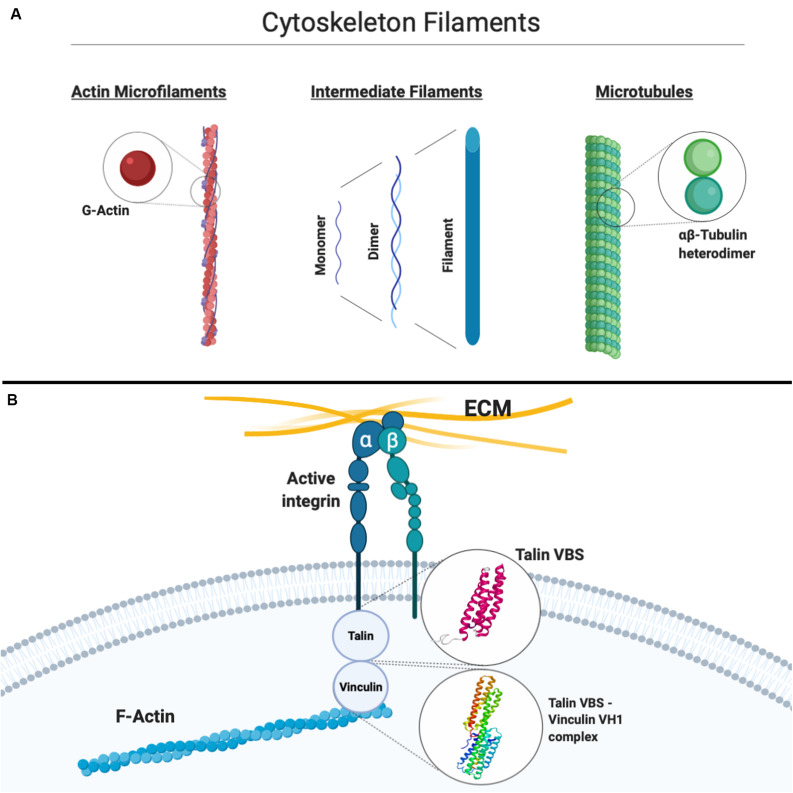
**(A)** Simplified schematic of the three main constituents of the cytoskeleton, that is, actin microfilaments, intermediate filaments, and microtubules, with their basic constitutive units highlighted. **(B)** Schematic representation of the connection between the extracellular matrix (ECM) and the actin cytoskeleton through integrins. Atomistic models available for the vinculin-binding site (VBS) of talin and the vinculin Vh1–talin VBS complex are shown. Created with Biorender.com.

The coupling between integrin receptors and the cytoskeleton sees the involvement, among others, of talin and vinculin (Figure 2). Moving further downstream of the mechanical chain connecting the cell environment to the inside of the cell, computational modeling has again proven to be a valuable tool in synergy with experimental approaches to unveil the causative factors of the translation of mechanical stimuli into competent or aberrant cellular responses. Talin, a globular protein crucial for linking integrins to the actin cytoskeleton, has been suggested through MD simulations to undergo a conformational change of its helical bundles when force is applied at the focal adhesion, effectively exposing cryptic binding residues crucial for vinculin binding (Lee et al., 2007), leading to the strengthening of focal adhesions, as shown by more recent works (del Rio et al., 2009; Yao et al., 2014). Vinculin, simultaneously binding to talin and F-actin, is also known to be essential for the maturation of stable focal adhesions. As Lee and co-workers suggested (Lee et al., 2007), the requirement of an external force to induce vinculin recruitment might be a crucial component in the overall mechanotransduction pathway. In their study, MD simulations were employed and a binding mechanism between the vinculin head subdomain (Vh1) and the talin vinculin-binding site (VBS) was proposed as characterized at the atomic scale in terms of consecutive structural events from hydrophobic insertion on VBS into Vh1 to a conformational rotation of the former (Lee et al., 2007). The predominantly hydrophobic nature of the VBS–vinculin interaction has been later confirmed again through MD simulations by Golji et al. (2011), further confirming the hypothesis of the insertion of the VBS of talin into the hydrophobic vinculin domain 1 core. Interestingly, the more general validity of the proposed mechanism suggested by Lee et al. (2008) constitutes a first example of how the understanding of molecular mechanisms in this context through computational models constitutes an important piece of knowledge, which might eventually lead, in tandem with experimental validation, to a broader multiscale model of mechanotransduction. Moreover, in the light of studies highlighting the importance of vinculin and in general of the cytoskeleton-binding proteome in heart contractility, cytoskeletal stiffening, and cardiac aging and disease (Heling et al., 2000; Kaushik et al., 2015), it is legitimate to expect that progress in the understanding of cardiac biomechanics can be effectively aided by computational, multiscale molecular models able to explain the functional and causative link between proper mechanotransduction and physiological function.

### Computational Investigation of Cytoskeleton Mechanics and Dynamics

Mechanotransduction involves a continuous dynamic rearrangement of the cytoskeleton as an instantaneous response to mechanical stimuli on the cell. Molecular mechanisms driving its activity are still not completely understood, but in the last decades, many experimental studies have been carried out in order to better understand how the cytoskeleton filaments behave under different stress conditions. Specifically, several studies in the past were purposed to shed light on mechanical properties of single cytoskeleton filaments (Figure 2), that is, MTs, which are polymers of hierarchically assembled tubulin heterodimers, actin MFs, which are polymers of G-actin, and IFs, which are polymers of intermediate diameter of around 10 nm, commonly divided into six classes on the basis of their composition (Cooper, 2007): computational approaches allowed to add valuable information to the experimental studies aimed at characterizing the biological cytoskeleton filament building blocks. These will be briefly described below.

Microtubules have been widely investigated by modeling approaches spanning from atomic level to continuum, with the common aim to shed light on the causative elements of their peculiar mechanical and electrical properties. To this regard, intracellular processes in the cell are thought to involve electric fields and, because MTs are polar structures and vibrate with frequencies of the order of MHz to GHz (Cifra et al., 2011, 2015), it has been hypothesized that MT charge motion produces electric fields that can interact with subcellular structures and organelles in such processes. In more detail, MTs have been suggested to be excellent conductors of electrical signals and able to amplify an electrical current and axial ionic movements (Priel et al., 2006) in virtue of pores in their lattice structure (Freedman et al., 2010). The latter’s proximity to negative-charge-bearing C-terminal tails further enables the interaction with ions, causing conduction through the lumen. Considering the essential role of electrical signal propagation for the cardiovascular function, the exploration of ionic currents through the MT might also be of interest in heart biomechanics. In terms of vibrational properties, the analysis of molecular reasons beneath MT mechanics and vibrations has been a highly considered research field in the scientific community. Molecular modeling has provided much information regarding this topic by synergistically coupling with classical continuum theories. There have been a number of computational studies dealing with MT elasticity, starting from one-dimensional models of the protofilament chain (Pokorný et al., 1997) to the development of hollow cylinders consisting of parallel beams, up to a two-dimensional lattice structure for the MT (Kis et al., 2002; Kasas et al., 2004a, b). A series of studies addressed the characterization of MT longitudinal vibrations (Pokorný et al., 1997; Kis et al., 2002; Kasas et al., 2004a, b; Deriu et al., 2010; Cifra et al., 2011; Barzanjeh et al., 2017). These models allowed to elucidate relationships between tubulin molecular structure and arrangement in the MT lattice and filament principal modes of motion. Vibration dispersion relations accounting for the geometrical architecture of MT as well as insights on protein–protein interactions were predicted in detail. Also, a strong dependence of the microscopic vibration properties of the MTs on the number of the MT protofilaments and MT lattice type has been found (Portet et al., 2005). By analyzing the normal modes of motion, it has been found that circumferential bending, controlled by circumferential bending stiffness, is dominant in circumferential vibrations. Therefore, the excitation of the vibrations could lead to a large deformation of the MT section, thus influencing both the mechanical performances and the structural integrity (Wang and Zhang, 2008; Zhang and Wang, 2014). Several theoretical models have been developed in order to justify experimental evidence demonstrating that the mechanical behavior of the MT could be better described by using the elasticity theory for transversally anisotropic structures (Kis et al., 2002; Kasas et al., 2004b). New insights about MT mechanical properties have been obtained by inspecting the MT mechanical characteristics starting from the atomic level, using a bottom-up approach. For example, anisotropic network model approaches have been able to shed light on the global dynamics of the tubulin dimer (Keskin et al., 2002) and even to predict vibration modes of entire MTs still maintaining an atomic resolution (Deriu et al., 2010).

Just as for MTs, computational investigations of the mechanical properties of actin were initially performed by considering actin filaments as homogeneous and isotropic rods (Yoshimura et al., 1984). However, soon, it has been understood that a potentially vast range of motions and flexibilities of the filaments remained elusive in this approach and that the isotropic rod model does not account for the hierarchical structure of actin filaments, composed of a double helix of G-actin polymers. This was corroborated by the observation of the peculiar 3D atomic models of the actin filament with its unique structural features (ben-Avraham and Tirion, 1995), again showing how the atomistic-scale bottom-up approach yields enhanced insights into mechanical characteristics. At the same time, owing to the huge computational cost of modeling cytoskeleton filaments while keeping their atomic structure into account, CG approaches have been introduced for the same purpose (Deriu et al., 2011, 2012; Bidone et al., 2015). One of the first MD simulations of G-actin in explicit solvent was performed by Wriggers and Schulten (1997), providing an understanding of actin dynamics and functional activity and revealing a propensity of G-actin to alter its structural conformation through a modification of the nucleotide binding cleft. It was soon understood that the application of mechanical stress does indeed play a role in the kinetics of G-actin and thus in the transduction of mechanical stimuli to the cytoskeleton. Lee and colleagues (Lee et al., 2019) recently determined experimentally how the history of the applied forces, especially cyclic mechanical stress, conditions the G-actin–G-actin and G-actin–F-actin association and dissociation kinetics and strengthens the inter-dimer contacts between G-actin dimers, leading to a mechanical reinforcement, thus acting as a possible mechanotransduction mechanism that allows for the formation of stable cytoskeletal networks as a result of a form of mechanical conditioning. Moreover, SMD studies provided an insight on how the application of force can induce the formation of salt bridges between G-actin subunits, hence significatively altering G-actin dynamics (Lee et al., 2013). Studies employing a CG approach (Chu and Voth, 2005, 2006) allowed to understand how protein conformational changes may significantly modify the inter-monomer interactions of actin assemblies, leading to wider, shorter, and more disordered filaments. Other computational approaches coupled microscopic- and macroscopic-scale levels to predict actin polymerization-related phenomena such as protrusive forces for the directional motion of a virtual lamellipodium. Modeling approaches also allowed to highlight how very stiff MFs are able to generate protrusive force also with a low concentration of G-actin monomer, whereas non-stiff MFs can only generate a protrusive force when enough G-actin monomers are supplied for actin polymerization. Collective long-range deformations of a filament, such as bending, twisting, and stretching, were investigated by NMA-based approaches useful to get information on principal components of the filament motion (ben-Avraham and Tirion, 1995).

Lastly, properties of IFs and IF-associated proteins were investigated by molecular modeling and often by SMD simulations, a powerful technique to characterize protein mechanics. The transition of the spectrin α-helix domain from a folded to an unfolded arrangement in response to the application of a tensile load was discussed in several computational works (Paramore and Voth, 2006). Moreover, in the light of study findings on the capability of IFs (especially keratin, vimentin, and desmin), to sustain large deformations above 250% (Herrmann et al., 2007; Kreplak et al., 2008), the tensile properties of IFs were investigated through computational models specifically on the vimentin dimer using SMD to evaluate the mechanical response to strain and link the macroscopic mechanical properties to structural and geometrical changes in the dimer (i.e., its unfolding state) with atomic resolution (Qin et al., 2009). This is but one example of how computational models can elucidate the key structural features responsible for the final mechanical behavior, with good agreement with experimental data.

## Mechanosensing in Cardiac Disease

As mentioned above, the mechanosensing machinery plays a fundamental role in the development and maturation of the heart as well as its physiological function. In the healthy heart, cardiomyocytes (CMs) are exposed to the passive stiffness of the cardiac muscle (independent of the muscle activity) and to the active force of the heart contractile apparatus. In cardiac disease, the active force of the heart changes owing to the loss of CMs or the expression of motor proteins together with changes in the cardiac load and output (Roca-Cusachs et al., 2012; Andres-Delgado and Mercader, 2016). Two different modeling strategies for CMs mechanotransduction have been proposed. The “localized” model involves the generation of a stretch signal in close proximity to the plasma membrane, whereas the decentralized model involves forces generated at the cell surface, which are transmitted via the cytoskeleton to other part of the cells (Tavi et al., 2001; Knoll et al., 2003). The generation of a stretch signal near the cell may involve non-selective ion channels that can be activated by longitudinal stretch of the cell. These channels induce an increase in cations (Na^+^ and Ca^2+^), which cause a release of Ca^2+^ from the sarcoplasmic reticulum, thereby acting as a transducer of a mechanical force into a biochemical signal. Another, and probably the most known, stretch sensor in CMs involves the cytoskeleton and allows the process of mechanotransduction to occur at a different site from where the strain is applied (Ingber, 2003a, b). These models are supported by *in vitro* studies where it has been shown that the application of a mechanical stimulus to isolated CMs or stem cells results in changes in gene and protein expression that are indicative of a (patho)physiological response or cell maturation and differentiation (Vining and Mooney, 2017; Kim et al., 2018; Wong et al., 2018). A possible mechanism for this involves many different proteins ranging from the ECM molecules to membrane and intracellular proteins that bind to the cytoskeleton and orchestrate the cellular response to different microenvironments. The ECM is fundamental in providing mechanical signals to the cells through different integrin receptors, which bind to the focal adhesion complex (FAC) and connect integrin receptors to the cytoskeleton (Belkin and Koteliansky, 1987; Bois et al., 2006; Ye et al., 2014). During development, the expression of the ECM in the developing cardiac tissue is tightly regulated, and several studies, using different animal models, have highlighted the ECM dynamics during embryonic development and in the adult heart. Briefly, collagen I is predominant in both developing and adult tissue, but in the latter, its density and cross-linking are upregulated, and the ratio between collagen I and collagen III decreases (Thompson et al., 1979; Borg, 1982; Hanson et al., 2013; Gonzalez et al., 2019). Fibronectin is highly expressed during embryonic development and is downregulated in the adult tissue (Kim et al., 1999; Lockhart et al., 2011). Conversely, elastin expression has been found to increase in the adult heart (Kim et al., 1999). These changes are generally associated with an increase in cardiac stiffness, with the heart tissue becoming less compliant during heart development, and CM differentiation. In CMs, the cytoskeleton is also anchored to the sarcomeric Z-disk and the myosin protein is stabilized by titin and other structural proteins, suggesting that the sarcomeric proteins also play an important role in CM mechanotransduction (Frank and Frey, 2011; Buyandelger et al., 2014). Several cardiac diseases are characterized by altered mechanical load and are characterized by morphological changes of the ventricular tissue and myocyte shape (Gerdes and Capasso, 1995). Dilated cardiomyopathies are characterized by volume overload and eccentric hypertrophy in which the sarcomere proteins are added in series, resulting in elongated myocytes. These changes ultimately lead to dilation of the ventricular chamber and thinning of the ventricular wall (Grossman et al., 1975; Beltrami et al., 1995). Conversely, hypertrophic cardiomyopathies are characterized by a pressure overload and concentric myocyte hypertrophy in which sarcomeric proteins are added in parallel, causing an increase in cross-sectional area of the myocytes. These changes lead to reduction in the volume of the ventricular chamber, thickening of the ventricular wall, and diastolic dysfunction (Zierhut et al., 1991; Katz, 2002). Both changes in myocytes shape are ultimately associated with impaired contractility and heart failure due to the altered force produced by CMs, indicating the importance of the mechanosensory machinery in the development and progression of cardiac disease (Gjesdal et al., 2011; Wilson et al., 2014; Janssen, 2019). The maladaptive response of the myocytes are also associated with several genetic mutations in sarcomeric proteins (β-myosin heavy chain, troponin T, and troponin I) (Kamisago et al., 2000) or protein associated with force transmission to the ECM, such as dystrophin (Lapidos et al., 2004). Mutations in these proteins suggest that the proper interaction of the contractile apparatus or the mechanosensing machinery with the cytoskeleton could be altered, thus resulting in a maladaptive response of the myocytes to mechanical loading. Another important feature of most of the cardiomyopathies is ECM remodeling. This is evidenced by numerous studies reporting that both the composition of the ECM and the expression of different integrin receptors are altered in many cardiomyopathies, with ischemic or non-ischemic origins. The remodeling phase associated with myocardial infarction is characterized by an increase in fibronectin and collagen deposition, which ultimately leads to fibrosis (Frangogiannis, 2017) and to an increase in myocardial stiffness (Berry et al., 2006), and it is also associated with a differential expression of α1, α3, and α5 integrins in both acute and chronic stages of myocardial infarction (Nawata et al., 1999). Similar to ischemic cardiomyopathies, pressure overload and genetic-based cardiomyopathies are also characterized by fibrosis and altered expression of integrin levels (Samuel et al., 1991; Schaper and Speiser, 1992; Ross and Borg, 2001; Ross, 2002). Integrin receptors are important in many cellular physiological processes, including cytoskeletal remodeling, and are the main receptors associated with mechanical signaling interacting with various adhesion complex proteins. Their changes in response to the diseased environment affect the mechanical signaling pathway and can ultimately lead to disease progression. An important player in all the previously mentioned pathological features is the cytoskeleton. In consequence of altered mechanical stimuli, genetic mutation, or ECM remodeling, the cytoskeleton responds in different ways ranging from a diminished affinity of the cytoskeletal elements to their binding proteins, thus impairing a physiological response to the mechanical stress, to structural changes in the cytoskeleton following an increase mechanical stress resulting in changes in cell phenotype and reorganization of the sarcomeric apparatus (Sequeira et al., 2014). The understanding of the cytoskeletal remodeling and its adaptation to physiological/pathological stimuli is therefore of fundamental importance in order to better understand the contribution of the mechanosensing machinery to the development and progression of most cardiomyopathies, and it may open new potential therapeutic targets in numerous cardiac diseases.

## Mechanosensing in Tissue Regeneration and Modeling

With the development of new emerging technologies in tissue generation and the use of iPSC-derived CMs, the *in vitro* generation of cardiac tissue has become a valid option to re-create *in vitro* different models of healthy and pathologic cardiac tissue to use both for regenerative medicine application and for research purposes. In order to generate *in vitro* cardiac-like tissue, either in the form of 3D construct or in the form of organoids, a combination of soluble (e.g., growth factors) and physical cues is necessary to form a fully mature and functional tissue. The soluble cues have been extensively studied (Paige et al., 2015; Denning et al., 2016; van den Berg et al., 2016; Leitolis et al., 2019), whereas the role of the physical cues is only recently been explored as a valuable approach to induce fully the maturation of the generated tissue (Lindsey et al., 2014). Despite great improvements in the differentiation of human pluripotent stem cell-derived CMs (hPSC-CMs) using a variety of approaches, the main limitation for the experimental use of these cells still remains their immature phenotype (Lieu et al., 2013). As we mentioned previously, mechanical stretch and electrical activity are important factors in the development, maturation, hypertrophy, and disease progression of the heart and should be considered in order to generate a functionally mature phenotype of the hPSC-CMs. Among the physical factors that should be taken into consideration for the development of a fully mature cardiac tissue are the ECM or scaffold in which the cells are cultivated, its rigidity and topography, mechanical loading and stretch, shear stress, and electrical stimulation. The ECM plays a fundamental role in presenting both biochemical and physical signals. When the cells are cultured in a 3D environment, the scaffold in which they are cultivated provides both biochemical and physical signal as the cells feel their environment and pull it. We have previously mentioned that MSCs respond to the substrate stiffness and differentiate according to the compliance of the different tissues (Engler et al., 2006). Similarly, it has been shown that also embryonic stem cell (ESC) differentiation could be driven by the rigidity of the substrate in which they are cultivated (Evans et al., 2009), and it should be taken into consideration in the development of *in vitro* cardiac-like tissue. Another important aspect of the ECM component is the biochemical composition. A decellularized cardiac matrix has been shown to be advantageous in culturing progenitor cells as well as ESC-CMs (Williams et al., 2014, 2015; Gaetani et al., 2016; Bejleri et al., 2018), probably due to its complex biochemical composition. Similarly, Jung et al. (2015) identified fibronectin, laminin-111, and collagen type 1 signaling to be very important in driving the differentiation of murine-derived iPSC-CMs. In the human body, the ECM is mainly synthetized by the fibroblast in response to different stimuli. It has been shown that the differentiation of ESC or iPSCs-CMs in a 3D culture is enhanced when the cells are co-cultured with fibroblast (Liau et al., 2011; Ou et al., 2011; Valls-Margarit et al., 2019). The fibroblast plays an important role in maintaining and influencing CM function by secreting growth factors (Ottaviano and Yee, 2011), propagating the electrical impulse (Gaudesius et al., 2003; Ogle et al., 2016), and secreting ECM (Zhang et al., 2012; Ogle et al., 2016). In a 3D scaffold, mainly, if it is a hydrogel or soft gel with poor compliance than the heart tissue, the fibroblast can act by remodeling the ECM and allowing a more robust gel compaction and remodeling, which results in a better cell-to-cell contact and likely an increase in tissue mechanical properties, thereby enabling the mechanosensing-mediated signaling, which is fundamental for tissue maturation. Interestingly, in a recently published work, the authors reveal a unique gene signature defining specific quiescent CF and showed that co-culture of hPSC-CFs with hPSC-CMs alter the electrophysiological properties of the CMs, as compared with co-culture with dermal fibroblasts, indicating the importance of tissue specificity, and could be used not only to improve the maturation of cultured cells but also to reveal the specific molecular signaling that regulates, and is regulated by, mechanosensing (Zhang et al., 2019). Another important aspect in the generation of functionally 3D cardiac tissue is the perfusion, which allows enough nutrient supply to the cultured cells, and it overall results in better cell survival, cell organization, and tissue compaction (Carrier et al., 2002; Radisic et al., 2008). Several systems have also integrated electrical stimulation into a perfusion bioreactor showing that the electrical stimulation is important in driving cell maturation and allows synchronous contraction of the perfused construct (Radisic et al., 2008). Nunes et al. (2013) generated a platform named biowire by 3D culturing human iPSC (hiPSC) or ESC-CMs together with supporting cells into a template of polydimethylsiloxane (PDMS) channel, around a sterile surgical suture in type I collagen gels. After 1 week, the cells organized along the suture and were electrically stimulated with increasing intensity over time. The authors demonstrated that the combination of geometric control and electrical stimulation resulted in improved electrical and ultrastructural properties of human cardiac tissue, resulting in cell maturation (Nunes et al., 2013). Similar results were observed in another recent work. Ronaldson-Bouchard et al. (2018) used early stage iPSC-derived CMs, soon after the initiation of spontaneous contractions, and electrically stimulate them with increasing intensity over time. This approach allowed increased maturation of the iPSC-CMs, which showed a gene expression profile and cell ultrastructure, including sarcomeres, T-tubules, and mitochondria, similar to the adult myocytes (Ronaldson-Bouchard et al., 2018, 2019). Another physical stimulus that has been frequently used in cardiac tissue engineering application is mechanical stimulation that consists in the external application of a mechanical stretching apparatus. The applied tension can be static or cyclic, and the stretching can be uniaxial, biaxial, or multiaxial (Cao et al., 2015). The approach of mechanical stretch to induce myocyte maturation has been used for the first time almost two decades ago (Zimmermann et al., 2000) using neonatal rat ventricular myocytes, and the advantages of this pioneering work have been further confirmed by numerous other studies. More recently, when human ESC and iPSC-CMs were 3D cultured in collagen gels and subjected to static or cyclic mechanical stress; the 3D culture promoted a better differentiation of the seeded progenitors, and mechanical stretch influenced maturation and structure of the differentiated tissue (Ruan et al., 2015). In another recently published work, the authors combined mechanical stimulation with electrical stimulation showing that the mechanical stimulation increases cell morphology, alignment, and contractility, whereas additional electric pacing resulted in an additional increase in force production (Ruan et al., 2016).

As of now, we know very little about the molecular mechanisms involved in the maturation of the 3D tissue, as most of the studies aimed at the generation of functional tissue and their characterization. However, it is likely that the same mechanosensing pathways described previously allow also the maturation of the different cultured 3D tissues. Recently, it has been shown that following mechanical stretch, Piezo1, phosphorylated-Ak transforming serine473 (P-AKTS473), and phosphorylated-glycogen synthase kinase-3 beta serine9 (P-GSK3) expression was downregulated in the human CM cell line AC16 (Wong et al., 2018). In another study, the mechanical stretch induces endothelial nitric oxide synthase gene expression in neonatal rat CMs (Cheng et al., 2009). Understanding the molecular pathways associated with mechanical stimuli-mediated maturation of the tissue could be important not only in the generation of functional cardiac *in vitro* tissues but also in the generation of pathological models that resemble the human pathology and could be important in our understanding of the diseased tissue but also in finding a new molecular target for clinical applications.

## Conclusion and Future Perspective

Independently from the etiology, heart failure is the funnel neck of the balance between genetic and epigenetic (inflammatory and fibrogenic) specific factors involved in the tissue damage and the constant variables to which these factors need to match, that is, mechanosensing and mechanotransduction. With the development of new technologies and a better understanding of the different biochemical and physical stimuli that allow the development, growth, and physiological function of a tissue, it is becoming more realistic to re-create *in vitro* healthy or pathological tissue that can be used for regenerative medicine or drug screening purposes.

The investigation of the mechanics of proteins involved in cell–cell and cell–ECM contacts, from integrins and stretch-sensing ion channels to cytoskeleton filaments, has been a very widely investigated topic by both experimental and computational studies, given the high importance of shedding light on the ability of cells to respond to external stimuli and given the importance thereof in a variety of physiological functions, including in particular the biomechanics of cardiac tissue. Consequently, molecular and multiscale simulations can help experimental studies to shed light on the many debated issues concerning cell mechanics and the broader phenomenon of mechanotransduction. It is also clear that modeling shall not be a substitute of experimental studies but rather a valuable tool to add complementary information, ultimately helping the interpretation or rationalization of experimental results. The possibility to simulate a fraction of the cytoskeleton, for example, a network of interconnected MTs, MFs, and IFs, maybe even connected to transmembrane transducers such as integrins, seems now to be an explorable possibility. The refinement of methodologies combined with the fast growth of computational capabilities might leave us astonished in the upcoming future.

On the other hand, the discovery of iPSCs has represented a major development in the generation of healthy and diseased functional cells or tissue. The possibility to generate CMs from healthy and diseased patients allows us to generate tissue that can be used for replacement therapy or for drug screening or validation. hIPSC-CMs have been generated from numerous patients with inherited cardiomyopathies and have allowed us to better understand the molecular pathways responsible in the development of the pathological phenotype (Hoes et al., 2019). However, as mentioned previously, the canonical 2D culture and differentiation of these cells still has major limitations, most notably their immature phenotype, which lacks to fully represent developed tissue. The development of new biomaterials (Reis et al., 2016; Wissing et al., 2017; Kuraitis et al., 2019) and the emerging of new technologies such as tissue printing (Charbe et al., 2019; Tomov et al., 2019), organ on a chip (Marsano et al., 2016; Ugolini et al., 2018; Wan et al., 2018), and different types of bioreactors that allow mechanical, perfusion, or electrical stimulation (Freed et al., 2006; Lei and Ferdous, 2016; Paez-Mayorga et al., 2019) allow us to generate cardiac tissue that more closely recapitulate the (patho)physiological features of the developed myocardium. Moreover, these models represent an optimal tool not only to test and validate new drugs or to re-create tissue substitute for regenerative medicine application but also to allow a better understanding of the molecular mechanisms behind disease development and progression. This is particularly important for the mechanosensing machinery and the generation of biochemical signals from physical cues, which are very difficult to recapitulate in static 2D culture conditions. As mentioned previously, these mechanisms are at the basis of many, if not all, cardiomyopathies, whether inherited or not, and could potentially open up new approaches and discover potential new targets for the cure of these pathologies. However, numerous challenges still remain to be addressed, such as the differentiation state of the CMs, the proper stimuli, when and how apply them, and how to coordinate *in vitro* the plethora of the different biochemical and physical signals that are responsible *in vivo* for the development and physiology of the functional tissue.

## Author Contributions

All authors listed have made a substantial, direct and intellectual contribution to the work, and approved it for publication.

## Conflict of Interest

The authors declare that the research was conducted in the absence of any commercial or financial relationships that could be construed as a potential conflict of interest.
